# Prevalence and associated factors of undiagnosed atrial fibrillation among end-stage renal disease patients on maintenance haemodialysis: a cross-sectional study

**DOI:** 10.1186/s12872-020-01473-6

**Published:** 2020-04-21

**Authors:** Izzat AlAwwa, Reham Al-Hindi, Nadeen Alfraihat, Ahmad Obeid, Sarah Ibrahim, Shatha Jallad, Ahmad Al-Awwa, Akram Saleh

**Affiliations:** 1 School of medicine, the University of Jordan, PO Box 954180, Amman, 11954 Jordan; 2 the University of Jordan, Amman, Jordan; 3 Al-Essra Hospital, Amman, Jordan; 4 King Hussein Medical Centre, Amman, Jordan

**Keywords:** Atrial fibrillation, Nonvalvular, Arrhythmia, End stage renal disease, Haemodialysis, Stroke, Cardiovascular risk, Subclinical, Anticoagulation

## Abstract

**Background:**

Atrial fibrillation (AF) is the most prevalent sustained arrhythmia worldwide and it aggravates cardiovascular morbidity and mortality; however, this is largely under-diagnosed. Moreover, among end-stage renal disease patients on haemodialysis, AF is substantially more common and serious. The researchers conducted this study to assess the prevalence of, and the factors correlated with AF in Jordanian haemodialysis patients.

**Methods:**

In a cross-sectional analysis conducted from October 2018 to February 2019 in four tertiary hospitals, the researchers enrolled all consenting patients aged 18 years or older who were on haemodialysis for at least three months prior to the study. We screened for AF clinically by pulse palpation, precordial auscultation, by an automated blood pressure monitor and an electrocardiogram. The researchers reported qualitative variables as counts and frequencies, while continuous variables were summarised using the mean or median where necessary. We used multiple logistic regression with backward selection to identify independent risk factors of AF.

**Results:**

A total of 231 patients were enrolled; mean age was 54.8 ± 15.6 years (from 20 to 86), and 44.3% of them were women. The prevalence of AF was found to be 7.8% (95% CI, 4.8–12.2), with no gender disparity. Age (adjusted odds ratio [AOR] = 1.05; 95% CI, 1.01–1.10; *p* = 0.031), history of ischaemic heart disease (AOR = 3.74; 95% CI, 1.09–12.34; *p* = 0.033), history of smoking (AOR = 0.15; 95% CI, 0.02–0.60; *p* = 0.019), and low interdialytic weight gain (AOR = 0.50: 95% CI, 0.25–0.91; *p* = 0.031) were independently correlated to AF.

**Conclusions:**

The prevalence of AF among patients on maintenance haemodialysis is high, but largely undiagnosed. AF is generally associated with advancing age, history of ischaemic heart disease, lower interdialytic weight gain, and history of smoking. We suggest routine check-up of AF in this high-risk group of patients as anticoagulant therapy if indicated may prevent serious complications. However, there is a need for large-scale cohort studies and for the creation of regional chronic kidney disease and dialysis registries in the Middle East region.

## Introduction

Atrial fibrillation (AF) is the commonest cardiac arrhythmia, and a global public health problem associated with high rates of hospitalisation, disability, and complications such as heart failure, cardiomyopathy, and cardio-embolic events like stroke [1–4]. The incidence and prevalence of AF have been on a rise globally especially in high-income European countries [1, 5]. In fact, about 33.5 million persons or 0.5% of the world’s population is estimated to have AF with age-adjusted prevalence rates of 596.2 and 373.1 per 100,000 in males and females, respectively. These prevalence rates in both genders were higher than previous values of 569.5 and 359.9 per 100,000 population reported in 1990 for men and women, respectively [5]. A similar increase in disability and mortality due to AF was also noted [5]. This rising prevalence of AF is in sync with the increasing cardiometabolic risk factors such as obesity, hypertension, diabetes, and an increasing population of elders globally [1].

It is estimated that between 11 and 13% of the world’s population is affected by chronic kidney disease (CKD); with the prevalence of stage 5 CKD estimated at about 0.1% [6]. Worsening kidney function has been associated with the risk of cardiovascular complications especially among patients on haemodialysis [7–9]. Cardiovascular complications significantly contribute up to 50% of all-cause mortality among patients with end stage renal disease (ESRD) on haemodialysis in the United States [10], with sudden cardiac death, arrhythmia, heart failure, and coronary artery disease reported as the main cardiovascular causes of mortality [8]. During haemodialysis, rapid fluctuations in haemodynamics and electrolyte concentrations, as well as the induction of hypoxemia increases the likelihood of developing arrhythmias [11]. Also, the incidence of AF increases after dialysis due to increase in cardiac dimensions [12–14]. Although arrhythmias developed during haemodialysis are mostly brief, asymptomatic, and self-limiting, they have been independently associated with higher mortality and cardiovascular events [15, 16]. The incidence of AF in patients with ESRD on dialysis varies between 6.6 to 20% and is 2 to 3 times higher than in the general population [17–20]. Furthermore, AF in patients with ESRD is associated with higher morbidity and up to 50% mortality rate in a cohort of patients with ESRD undergoing haemodialysis [19, 21, 22]. Despite the significant impact of AF in patients with ERSD, this remains a neglected topic in the scientific literature, especially in the Middle East in general and Jordan in particular, as a detailed PubMed search revealed no study on the prevalence, incidence, or mortality of AF in patients with ESRD in the region.

This study sought to evaluate the prevalence and factors associated with previously undiagnosed AF among patients with ESRD on maintenance haemodialysis. The information from the study is vital to enlighten practice in the Middle East and add to the limited literature on topic globally.

## Methods

### Study design and setting

This was a cross-sectional analysis conducted from October 2018 to February 2019, in four haemodialysis centres in Amman. Each dialysis unit serves about 50 to 150 patients and is managed by a nephrologist.

### Study population and sample size

All patients with ESRD undergoing haemodialysis were screened for eligibility and then invited to take part in the study. The researchers enrolled all consenting participants aged 18 years or older, diagnosed with ESRD and had received haemodialysis for at least three months prior to the study. We excluded patients with a previous diagnosis of arrhythmia or AF, and any psychiatric, psychological, or debilitating disorder which could prevent the patients from either being interviewed or understanding the questions.

Using the Cochrane’s formula below, we calculated a minimum acceptable sample size (n) of 167 participants.

$$ n=\frac{Z^2P\left(1-P\right)}{d^2} $$

z = Z statistics = 1.96 for a 95% confidence interval (CI); p = prevalence of subclinical AF among patients with ESRD on dialysis = 0.124 [18]; and d = margin of error which was considered to be 5% for this study.

Participants were consecutively enrolled in the study.

### Ethical consideration

The study protocol was approved by the ethical review boards (IRB) of the participating hospitals. All patients signed an informed written consent form after being explained the details of the research, its procedure, the potential benefits and harm, and their questions were satisfactorily answered.

### Study procedure and data collection

Patients were interviewed by trained final year medical students using a pre-designed questionnaire to collect information on the general demographic information (e.g. age, gender, marital status, and occupation), past medical history (e.g. Family history of hypertension, history of diabetes mellitus, and ischaemic heart disease), and dialysis related information (e.g. duration of dialysis, dialysis access and number of sessions per week). Information on the patient’s drugs and results of the laboratory tests were also collected from the patient’s medical file. The patient’s body mass index was computed using the patient’s weight (measured in the nearest 10 g) and height (in centimetres). An automated blood pressure (BP) monitor with irregular heartbeat detector (Omron M6, Omron Healthcare Co. Ltd., Japan) was used to detect the presence of irregular heartbeat during BP recording. This device has been clinically validated for detecting AF during blood pressure recording with a sensitivity of 98.7% [18].

At baseline, prior to haemodialysis, we screened all patients for AF by palpating their pulse and auscultating their heart to check for irregularities using the automated BP monitor and a 12-lead electrocardiogram (ECG). The patients’ BP and pulse were also monitored during dialysis using the automated BP monitor. An irregular heartbeat sign on the device screen would flash if an irregular heartbeat was detected. If the sign flashed, the BP was remeasured after five minutes to confirm the presence of the sign. A second ECG was obtained if the sign persisted. Only patients with an episode of AF on 12-lead ECG were confirmed to have AF. All ECGs were performed by trained final year medical students, and the ECG findings were interpreted blindly by a consultant cardiologist in two separate sessions.

### Statistical analysis

We used Microsoft Excel 2010 spreadsheets for data entry and curation, and the statistical software R (version 3.5.3, The R Foundation for statistical computing, Vienna, Austria) for data analysis. We reported qualitative variables as counts and proportions while we summarized quantitative variables as means and medians with their corresponding standard deviation (SD) and interquartile range (IQR), respectively. Histograms and probability distribution plots were used to visually assess continuous variables for normality and presence of outliers. The Fisher’s exact test was used to compare categorical variables, while the student t-test or Mann-Whitney U test was used to compare continuous and categorical variables as appropriate. We determined independent factors correlated with AF using multiple logistic regression analysis utilizing backward elimination method [23]. Only variables with *p*-values less than 0.1 on bivariate analysis, with the exception of gender, were evaluated using backward elimination method and based on the Akaike information criterion (AIC) for final inclusion in the multiple regression model [23]. Lower AIC signified a better model. The best model (based on the AIC) constituted of the variables: age, history of smoking, average weight gain during dialysis, history of ischaemic heart disease, and diastolic blood pressure before dialysis. Two-tailed *p*-values below 0.05 were considered statistically significant.

## Results

### Description of the study population

Collectively, we enrolled 231 participants in this study. Only one candidate was excluded because of previous diagnosis of AF. The mean age of the participants was 54.8 years (SD = 15.6) and participants who developed atrial fibrillation were significantly older than their counterparts without atrial fibrillation (*p* < 0.001), Table 1. About 44.2% of the participants were females and 71.7% were married. About 85% were Jordanians.

**Table 1:** Sociodemographic data of the study population

Variables	Total *N* = 231 (100.0%)	AF *N* = 18 (7.8%)	No AF *N* = 213 (92.2%)	*p*-value
Mean age in years (±SD)	54.8 ± 15.6	65.9 ± 15.6	53.9 ± 10.5	< 0.001*
Gender				0.453
Females	102 (44.2)	10 (55.6)	92 (43.2)	
Males	129 (55.8)	8 (44.4)	121 (56.8)	
Marital Status				0.092
Married	165 (71.7)	16 (88.9)	149 (70.0)	
Single	65 (28)	2 (11.1)	64 (30.0)	
Nationality				0.674
Jordanian	200 (86.6)	15 (83.3)	185 (86.9)	
Others	31 (13.4)	3 (16.7)	28 (13.1)	
Body mass index (kg/m^2^) * (±SD)	26.0 ± 5.6	26.2 ± 5.6	26.0 ± 5.6	0.914

*Mean ± SD; AF = atrial fibrillation; SD = standard deviation

Three-quarters of the participants were hypertensive, almost 40 and 30% had diabetes mellitus or dyslipidemia, 14% had ischaemic heart disease while the presence of vulvulopathies were rare, Table 2.

**Table 2:** Past Medical History of the participants

Variables	Total N = 231 (100.0%)	AF N = 18 (7.8%)	No AF N = 213 (92.2%)	*p*-value
Hypertension, Yes (%)	174 (75.3)	15 (83.3)	159 (74.6)	0.412
Diabetes mellitus, Yes (%)	91 (39.4)	10 (55.6)	81 (38.0)	0.143
Dyslipidaemia, Yes (%)	68 (29.9)	6 (33.3)	63 (29.6)	0.738
Ischemic heart disease, Yes (%)	33 (14.3)	6 (33.3)	27 (12.7)	0.016*
Heart failure, Yes (%)	34 (14.7)	5 (27.8)	29 (13.6)	0.103
Valvulopathy, Yes (%)	4 (1.7)	1 (5.6)	3 (1.4)	0.195
Family history of dialysis, Yes (%)	48 (20.8)	3 (16.7)	45 (21.1)	0.654
History of smoking, Yes (%)	87 (37.7)	2 (11.1)	85 (39.9)	0.015*
Consume alcohol, Yes (%)	5 (2.2)	0 (0.0)	5 (2.4)	0.509
Tea intake, Yes (%)	182 (79.1)	16 (88.9)	166 (78.3)	0.289
Number of glasses of tea per week^&^	7 (2–14)	10.5 (7.0–19.3)	7.0 (1–14)	0.140
Coffee intake, Yes (%)	167 (72.6)	11 (61.1)	156 (73.6)	0.388
Number of cups of coffee per week^&^	7 (1–14)	7 (0–7)	3 (0–14)	0.153

^&^Median (interquartile range); *Meaan ± SD; AF = atrial fibrillation

On an average, the participants had three sessions of dialysis per week, with each session lasting four hours. Over three-quarters of the route of access for dialysis was an arteriovenous fistula. Participants gained an average of 3.1 kg in between the dialysis sessions, Table 3.

**Table 3:** Haemodialysis variables of participants

Variables	Total N = 231 (100.0%)	AF N = 18 (7.8%)	No AF N = 213 (92.2%)	*p*-value
Duration of dialysis (in hours) ^&^	4 (3.0–4.0)	3.5 (3.0–4.0)	4.0 (3.0–4.0)	0.260
Number of sessions per week ^&^	3 (3–3)	3 (3–3)	3 (3–3)	0.230
Dialysis access type				0.445
Arteriovenous fistula Yes (%)	177 (76.6)	12 (66.7)	161 (77.4)	
Arteriovenous graft Yes (%)	3 (1.3)	0 (0.0)	3 (1.4)	
Central dialysis catheter Yes (%)	51 (22.1)	6 (33.3)	44 (21.2)	
Pre-dialysis				
Average Systolic BP (in mmHg) *	133 ± 28.4	126.9 ± 34.5	133.6 ± 27.8	0.436
Average Diastolic BP (in mmHg) *	78.3 ± 13.5	71.3 ± 13.5	78.9 ± 13.4	0.034*
Post dialysis				
Average SBP (in mmHg) *	120.5 ± 28.6	121.0 ± 32.4	120.5 ± 28.3	0.950
Average DBP (in mmHg) *	73.3 ± 14.1	73.2 ± 14.5	73.3 ± 14.1	0.967
Average weight gain during dialysis (in kg) * (±SD)	3.1 ± 1.0	2.6 ± 1.0	3.1 ± 1.0	0.031*

^&^Median (interquartile range); *Mean ± SD; AF = atrial fibrillation; BP = blood pressure; SD = standard deviation

About 90, 70, and 40% of the participants were on calcium, alpha 1 hydroxylase vitamin D, and beta-blockers. About 23% were on furosemide, Table 4.

**Table 4:** Drugs used by participants and relevant lab results

Variables	Total N = 231 (100.0%)	AF N = 18 (7.8%)	No AF N = 213 (92.2%)	*p*-value
Omeprazole, Yes (%)	79 (34.2)	4 (22.2)	75 (35.2)	0.392
Lansoprazole, Yes (%)	75 (32.5)	6 (33.3)	69 (32.4)	0.935
Beta-blocker, Yes (%)	94 (40.7)	5 (27.8)	89 (41.8)	0.362
Calcium channel blocker, Yes (%)	100 (43.3)	4 (22.2)	96 (45.1)	0.102
ACE inhibitor, Yes (%)	10 (4.3)	1 (5.6)	9 (4.2)	0.790
Furosemide, Yes (%)	53 (22.9)	4 (22.2)	49 (23.0)	0.940
Calcium carbonate, Yes (%)	209 (90.5)	16 (88.9)	193 (90.6)	0.811
Vitamin D, Yes (%)	26 (11.3)	4 (22.2)	22 (10.3)	0.252
Erythropoietin, Yes (%)	77 (33.3)	6 (33.3)	71 (33.3)	1.000
Alpha 1 hydroxylase vit D, Yes (%)	163 (70.6)	15 (83.3)	148 (69.5)	0.333
Labs				
Low haemoglobin (anaemia), Yes (%)	21 (9.1)	1 (5.6)	20 (9.4)	0.891
Serum Ca^2+^, Low (%)	76 (32.9)	6 (33.3)	70 (32.9)	0.922
Serum K^+^, Low (%)	7 (3.0)	2 (11.1)	5 (2.4)	0.223
Hypoalbuminemia, Yes (%)	34 (14.7)	2 (11.1)	32 (15.0)	0.885

AF = atrial fibrillation; ACE = Angiotensin converting enzyme

### Prevalence of atrial fibrillation in the study population

Eighteen out of the 231 participants were diagnosed with atrial fibrillation giving a prevalence of 7.8% (95% CI, 4.8–12.2) with no gender disparity (*p* = 0.453), Table 1.

### Factors associated with atrial fibrillation

The final multiple regression model predicted age (adjusted odds ratio [AOR] = 1.05, 95% CI, 1.01–1.10, *p* = 0.031), ischaemic heart disease (AOR = 3.74, 95% CI, 1.09–12.34, *p* = 0.033), history of smoking (AOR = 0.15, 95% CI, 0.02–0.60, *p* = 0.019), and low interdialytic weight gain (AOR = 0.50, 95% CI, 0.25–0.91, *p* = 0.031) as independent factors associated with the occurrence of atrial fibrillation with a very good accuracy (C statistics = 82.2%), Table 5. As shown by the Nagelkerke pseudoR2 value, 25.4% of the variation in the outcome variable was explained by the aforementioned independent variables.

**Table 5:** Bivariate and multiple logistic regression analysis of factors associated with subclinical atrial fibrillation

Variables	OR (95% CI)	*p*-value	Adjusted OR (95% CI)	*p*-value
Sociodemographic data				
Age in years	1.07 (1.03–1.12)	0.003*	1.05 (1.01–1.10)	0.031*
Gender, Female^&^	1.64 (0.86–4.35)	0.323		
Marital Status, Single	0.30 (0.05–1.08)	0.111		
Body mass index (BMI) (kg/m^2^)	1.00 (0.92–1.09)	0.912		
Past Medical History				
Hypertension, Yes (%)	1.70 (0.53–7.51)	0.417		
Diabetes mellitus, Yes (%)	2.04 (0.77–5.54)	0.151		
Dyslipidaemia, Yes (%)	1.19 (0.40–3.21)	0.738		
Ischemic heart disease, Yes (%)	3.44 (1.12–9.68)	0.022*	3.74 (1.09–12.34)	0.033*
Heart failure, Yes (%)	2.44 (0.74–7.02)	0.113		
Valvulopathy, Yes (%)	4.12 (0.20–34.20)	0.231		
Family history of dialysis, Yes (%)	0.75 (0.17–2.39)	0.655		
History of smoking, Yes (%)	0.19 (0.03–0.68)	0.029*	0.15 (0.02–0.60)	0.019*
Consume alcohol, Yes (%)	7.31e −07 (NA)	0.989		
Tea intake, Yes (%)		0.300		
Number of glasses of tea per week	1.01 (0.97–1.05)	0.522		
Coffee intake, Yes (%)	0.56 (0.21–1.60)	0.260		
Number of cups of coffee per week	0.95 (0.88–1.01)	0.164		
Dialysis				
Duration of dialysis (in hours)	0.57 (0.20–1.61)	0.292		
Number of sessions per week	1.06 e07 (NA)	0.992		
Dialysis access type				
Arteriovenous fistula	Ref			
Arteriovenous graft	8.57 e–07 (NA)	0.992		
Central dialysis catheter	1.83 (0.61–5.00)	0.253		
Pre-dialysis				
Average systolic BP (in mmHg)	0.99 (0.97–1.01)	0.340		
Average diastolic BP (in mmHg)	0.95 (0.92–0.99)	0.024*	0.96 (0.92–1.00)	0.078
Post dialysis				
Average systolic BP (in mmHg)	1.00 (0.98–1.02)	0.943		
Average diastolic BP (in mmHg)	1.00 (0.96–1.03)	0.966		
Average weight gain during dialysis (in kg)	0.55 (0.31–0.94)	0.035*	0.50 (0.25–0.91)	0.031*
Labs				
Low haemoglobin (Anaemia), Yes (%)	0.59 (0.03–3.13)	0.613		
Serum calcium, Low (%)	0.95 (0.32–2.56)	0.922		
Serum potassium, Low (%) ^&^	0.21 (0.04–1.59)	0.081		
Hypoalbuminemia, Yes (%)	1.48 (038–9.79)	0.621		

^&^Excluded during the backward elimination procedure; BP = Blood pressure; CI = confidence interval; OR = Odds ratio. Predictive power of final model: Nagelkerke pseudoR^2^ = 0.254; C statistics = 82.2%

Goodness-of-fit: Hosmer-Lemeshow test (X^2^ = 3.31, *p* = 0.913).

**Fig. 1:**
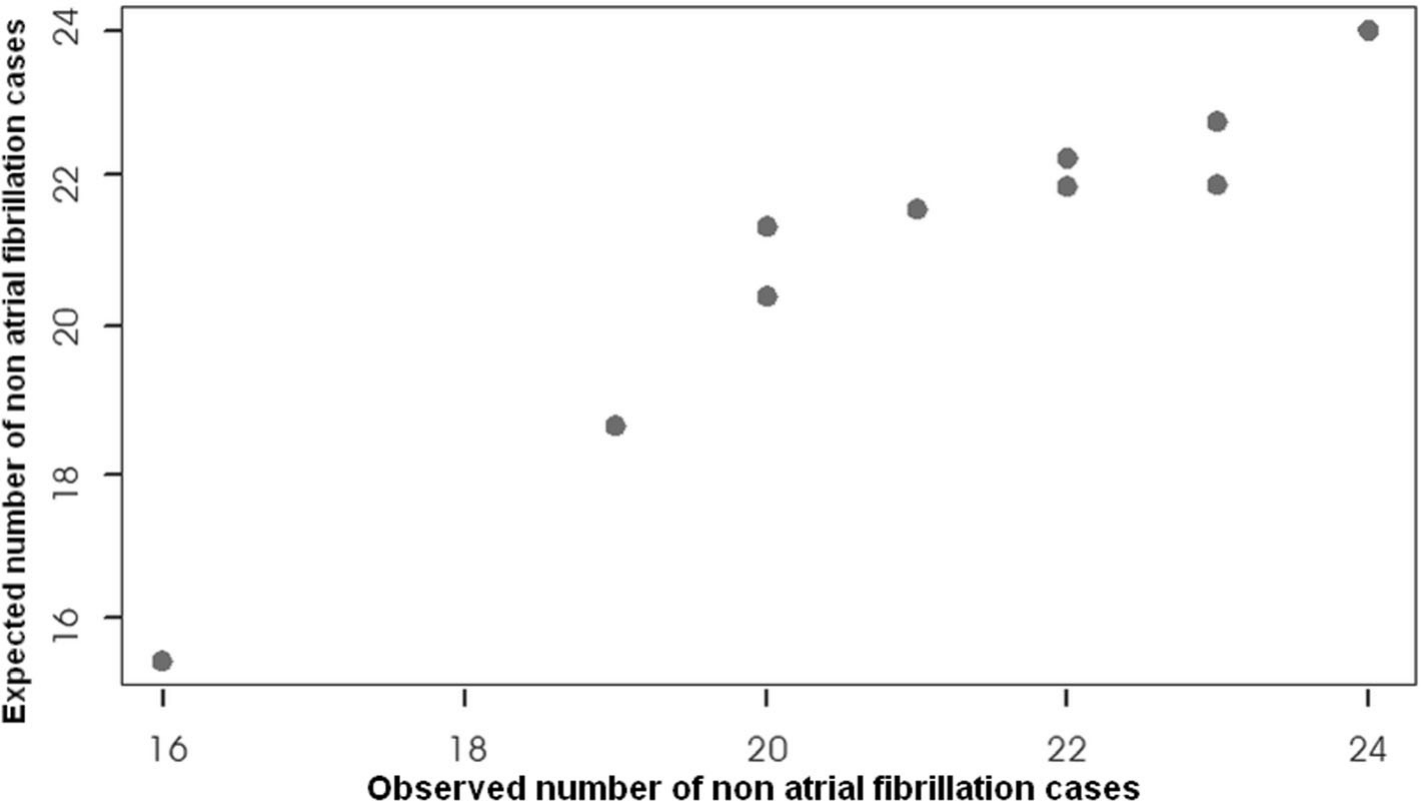
Observed vs. expected probability of non-atrial fibrillation cases Description: Hosmer-Lemeshow goodness-of-fit test. The plotted points follow a linear relationship, which testifies a good-fit of the prediction model of data

**Fig. 2:**
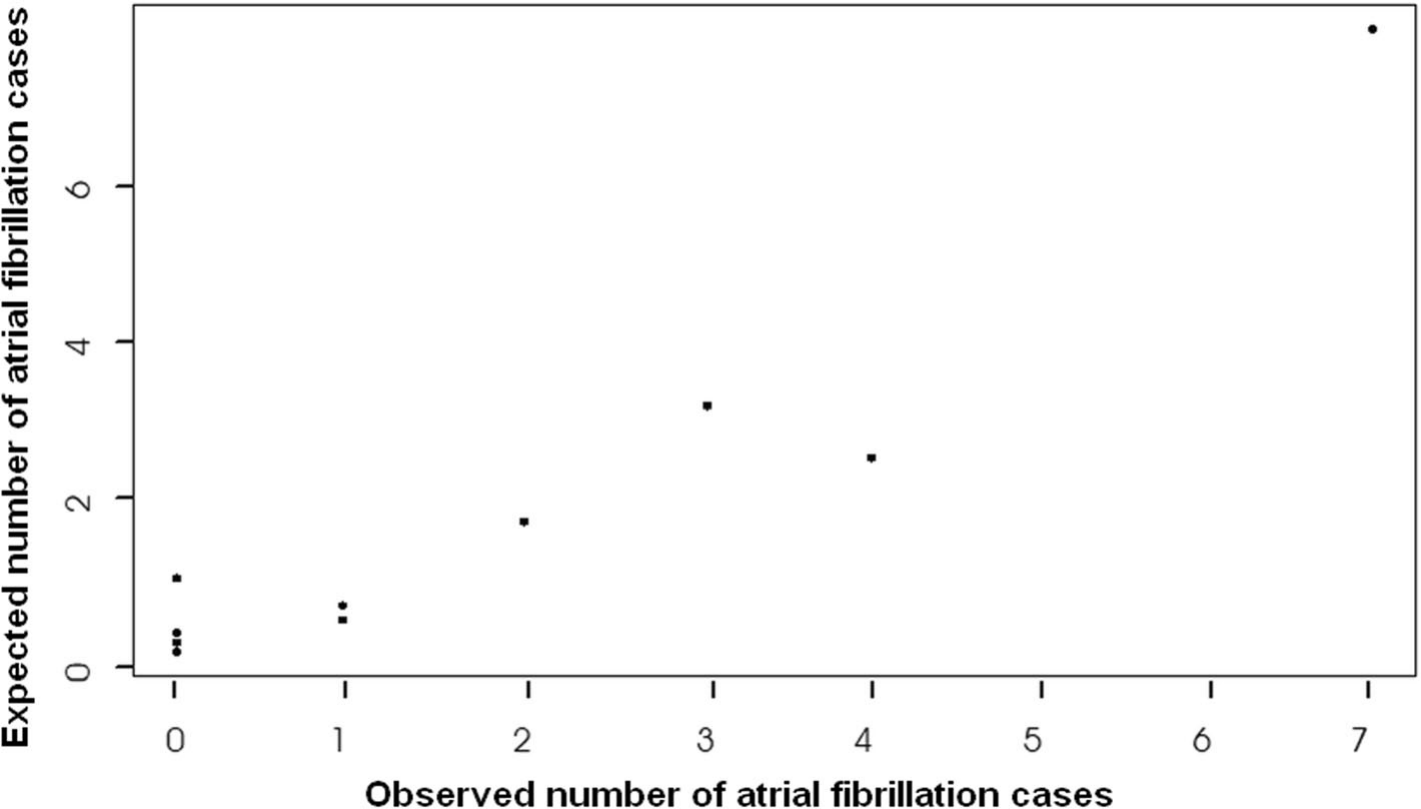
Observed vs. expected probability of atrial fibrillation cases Description: Hosmer-Lemeshow goodness-of-fit test. The plotted points follow a linear relationship, which testifies a good-fit of the prediction model of data

The model was also a good fit for our data as was revealed by a non-significant Hosmer-Lemeshow test (Chi-square = 3.31, *p* = 0.913), Table 5. Figures 1 and 2 display a good fit between the predicted data for both cases of atrial fibrillation and non-atrial fibrillation cases.

## Discussion

We sought to evaluate the prevalence and factors associated with atrial fibrillation among ESRD on maintenance chronic haemodialysis. The high prevalence of 7.8% was linked to increasing age, history of smoking, history of ischaemic heart disease and lower interdialytic weight gain with a very good accuracy.

Very few studies have evaluated the prevalence of subclinical AF among ERSD patients on maintenance haemodialysis. The prevalence of AF in this study is similar to rates of 7.7 and 8.0% reported by Konigsbrugge et al., 2017 [17] and Winkelmayer et al., 2011 [20] though much lower than those reported by Vazquez et al., in 2009 [18]. The lower prevalence in our study compared to that of Vasquez and colleagues is most likely due to the younger age of participants in our study as Vazquez et al. only recruited elderly participants (65 years or older). In addition, this could also be due to methodological differences as these studies were based on medical records contrary to our study as previously known cases of AF were excluded and we also depended on direct patient contact and an electrocardiogram to establish the diagnosis of subclinical AF. Most of our participants were within their fifth and sixth decades of life. This is a striking finding as it is the peak age for economic productivity and may therefore have a negative impact on the socioeconomic burden of ESRD in Jordan.

In the current study, age, history of smoking, history of ischaemic heart disease, and low interdialytic weight gain explained up to 25.4% of the total variability in the outcome. Age has been consistently reported in literature to be a strong predictor of AF [17, 18, 20, 24]. Aging is a normal physiological process which increases the individual’s risk of cardiovascular diseases such as hypertension, ischaemic heart diseases, and other comorbidities [24, 25]. These diseases are major risk factors for stroke. However, advanced age can directly cause AF even though the mechanism is still unclear [24]. However, existing theories suggest that advancing age leads to AF through atrial fibrosis, which is a major cause of atrial fibrillation [26].

Additionally, ischaemic heart failure is a well-established risk factor for AF [27]. A previous ischaemic heart disease will lead to cardiac remodeling, which predisposes the patient to aberrant intra-atrial conduction and consequently, AF [26]. Other cardiovascular disease risk factors such as heart failure and valvulopathy were not significant in our study [17, 18, 20, 24]. This is most likely due to the small number of participants with these comorbidities, Table 2.

The association between cigarette smoking and AF is controversial. Although a link has been demonstrated between smoking, mostly current smoking and AF in most part of literature, most studies conducted on ESRD patients on haemodialysis have failed to identify any associations [17, 18, 22, 25, 28]. Smoking was paradoxically protective against AF in our study. This finding was similar to those of Mariscalco and Engström who reported a protective effect of current smoking among participants who had undergone a coronary artery bypass and valvular surgical procedure [29]. However, a review by D’Alessandro et al. (2011) instead found smoking cessation to be preventive against AF [30]. A possible explanation for this association is that with chronic kidney disease being a chronic inflammatory state, ex-smokers whose cardiomyocytes have been exposed to higher adrenergic states can better tolerate the stress induced by chronic kidney disease and dialysis. However, this association is likely to be more complex and multifactorial, and there warrant further explorations.

Finally, in our study, lower interdialytic weight gain was associated with the occurrence of AF. Traditionally, higher interdialytic weight gain is linked to adverse morbidity and all-cause mortality and has been a growing area of interest in dialysis medicine [31–33]. However, the association between interdialytic weight gain and AF is poorly established as this has been poorly studied in literature. Even though increasing interdialytic weight has been associated with cardiovascular morbidity such as ischaemic heart disease [34], little or nothing is known about its association with AF. This association is likely linked to the inverse correlation of interdialytic weight gain with age. That is, patients with higher interdialytic weight gain tend to be younger [31] and less likely to develop AF [17, 18, 20, 24]. Nevertheless, the association between interdialytic weight gain and AF remained true even after controlling for age. In one study, dialysis patients who were hypertensive due to hypervolemic state were found to have lower interdialytic weight gain which may partially explain the higher AF rate in these patients [35]. Additionally, a small group of patients with low interdialytic weight gain, especially those with no residual kidney function, were found to have protein-energy wasting, malnutrition, or intercurrent illness that may explain a higher AF rate [33].

### Study limitations

Although we attained the minimum acceptable required sample size for this study, a larger sample size would have enabled us to detect smaller effects. Smaller sample sizes seem to be a major challenge for studies where data was collected prospectively using a cross-sectional design [18]. Most large-scale studies have been designed as either retrospective (using medical records) or prospective cohort. Also, despite the strict selection criteria including only patients without a prior diagnosis of AF, there is a possibility that we missed cases of paroxysmal AF. This study has several strengths. This is the first study on the subject in Jordan. Also, we established the diagnosis of AF based on recommended criteria. Furthermore, we used recommended and robust analytic techniques to build our regression model to make predictions with very good degree of accuracy.

## Conclusions

The prevalence of subclinical atrial fibrillation among ESRD patients on maintenance dialysis was found to be high and this was mainly driven by advancing age, history of ischaemic heart disease, history of smoking, and lower interdialytic weight gain. ESRD is distinctive in Jordan because it affects mostly younger individuals at the prime of their economic productivity, thereby posing a significant socioeconomic burden. We suggest checking routinely for AF in patients on haemodialysis and if present to consider anticoagulant therapy as this may prevent serious complications. Finally, more large-scale studies are required on the national territory and the Middle East region, with the creation of national and regional registries for prospective observations.

## Data Availability

The datasets used and/or analysed during the current study are available from the corresponding author on reasonable request.

## Abbreviations

AF: Atrial fibrillation
CI: Confidence intervals
OR: Odds ratio
AOR: Adjusted odds ratio
CKD: Chronic kidney disease
ESRD: End stage renal disease
BP: Blood pressure
ECG: Electrocardiogram
SD: Standard deviation
IQR: Interquartile range
AIC: Akaike information criterion
ACE: Angiotensin converting enzyme
